# 

**Published:** 1886-07

**Authors:** 


Entered at the Post Office at Austin, Texas, as Second-class Matter.

VOL. II.] JULY, 1886.

219

### DANIEL'S MEDICAL JOURNAL

*Subscription: $2.00 a Year in Advance.* *Single Copies, 25 Cents.*

F. E. DANIEL, M. D., Editor and Publisher, AUSTIN, TEXAS.
NORTHERN OFFICE, 1217 FILBERT STREET, PHILADELPHIA.

